# Evaluation of The Number of CD4^+^ CD25^+^ FoxP3^+^ *T*reg Cells in Normal Mice Exposed to AFB_1_ and Treated with Aged Garlic Extract

**Published:** 2013-05-05

**Authors:** Mohaddeseh Larypoor, Mansour Bayat, Mohammad Hassan Zuhair, Abbas Akhavan Sepahy, Masoud Amanlou

**Affiliations:** 1 Department of Mycology, Faculty of Veterinary, Science and Research Branch, Islamic Azad University, Tehran, Iran; 2 Department of Immunology, School of Medical Sciences, Tarbiat Modares University, Tehran, Iran; 3 Department of Microbiology, North Branch of Islamic Azad University, Tehran, Iran; 4 Department of Medicinal Chemistry, Faculty of Pharmacy and Medicinal Plants Research Center, Tehran University of Medical Sciences, Tehran, Iran

**Keywords:** Aflatoxin-B_1_, Garlic, *T*reg Cells, Immunotherapy

## Abstract

**Objective:**

Aflatoxin B1 (AFB_1_) suppresses the immune system. To decrease such suppressive effects on the immune system, a wide range of herbal medicines like garlic are utilized. Biological activities of garlic *in vitro* and *in vivo* have also been verified. Our previous studies demonstrated that aged garlic (dry garlic bulbs preserved in the freezer for six months at -20˚C) have increased immunostimulator fractions and reduced immunosuppressor fractions. This study focuses on the immunosuppressor activity of AFB_1_ and immunostimulator activity of aged garlic extract (AGE) through the evaluation of CD4^+^ CD25^+^ FoxP^+^ regulator cell (*T*reg) counts and the pattern of cytokine production in Balb/c normal mice.

**Materials and Methods:**

In this experimental research, AFB_1_ was separated from Aspergillus flavus (PTCC 5004) by HPLC and AGE prepared using the Mantis method. The Delayed-Type Hypersensitivity (DTH) test was carried out to determinate the effectiveness of different doses of AGE and AFB_1_, which can both have an effect on the immune system. Subsequent experiments were carried out on 20 Balb/c mice to estimate the effects of AGE and AFB_1_ on the number of *T*reg cell in 4 groups: 10 µl/kg/day of AFB_1_ and AGE diluents were administered for 4 consecutive days to group 1. AFB_1_, 2. control, 3. AGE + AFB_1_ and 4. AGE via intraperitoneal (IP) route, respectively. Mice were sacrificed and splenocytes harvested and the percentage of splenic *T*reg cells was measured by flow cytometry analysis. The ELISA method was utilized to measure Cytokine production.

**Results:**

The findings reveal that AGE increased the level of IFN-λ and IL-4 cytokines produced by splenocytes stimulated by specific tumor antigen and decreased the number of *T*reg cells in the spleen (p<0.05). AFB_1_ increased the number *T*reg cells in the spleen and decreased cytokine production (p<0.05). In groups 2 (control) and 4 (AGE) the number of *T*reg cells decreased (p value<0.05) whereas in groups 1 and 3 the number of *T*reg cells increased (p<0.05).

**Conclusion:**

This study indicated that AGE is able to alter the cytokine production in normal mice into a Th_1_ protective pattern which is beneficial to the immune system in general and anti-tumor immunity in particular. AFB_1_ is able to alter the cytokine production into a Th_2_ protective pattern. Therefore, AGE might be used as herbal medicine with few side effects as compared to chemotherapy in treating cancers caused by substances like AFB_1_.

## Introduction

**Fig 1:**
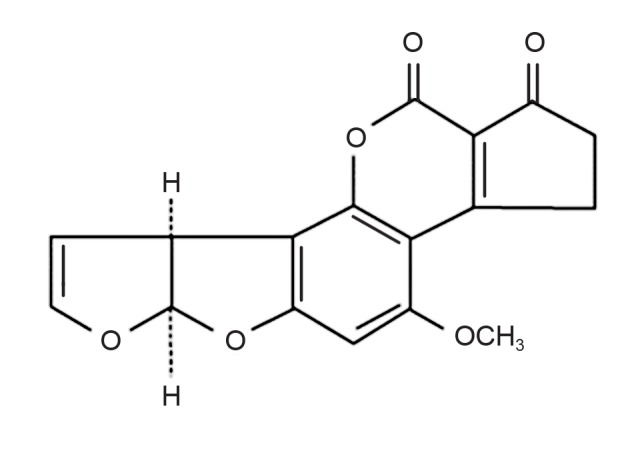
The structure of AFB_1_

AFB_1_, a secondary metabolite of the fungus *Aspergillus flavus*, is a hepatocarcinogen in various animal species, including fish, birds, rodents, and nonhuman primates (1-4). It is also a suspected human carcinogen and has been shown to play a role in human hepatocarcinoma (5-7). When low dosages of AFB_1_ are received on a daily basis over a long time, the number of *T*reg cells (CD4^+^ CD25^+^ FoxP3^+^) is changed in the human body (3,4). The authors present part of their immunotoxicity study, which aims to complement a larger, collaborative effort designed to assess potential biomarkers that may have a role in the initiation and promotional stages of carcinogenesis (9, 10), and which may be of relevance in the "risk assessment" process. In particular, the authors were interested in the effects of AFB_1_ on cells and the mechanisms of cell-mediated immunity (CMI) as this has been implicated as the immune target (11, 12) with respect to carcinogenesis. AFB_1_ has unique chemical structures, which cause harm by reacting with the chemicals in living organisms. The structure of AFB_1_ is shown in figure 1.

As a digestive stimulant, diuretic, and antispasmodic, garlic (Allium sativum, Liliaceae) is used by many people all over the world. Garlic has recently been reported to have antibiotic properties and benefits including antifungal (13) and antibacterial activities (14). It is also reported to have hypolipidemic, anti-atherosclerosis (15) and anti-carcinogenesis activities (16). Various research studies have indicated that garlic modulates immune responses (16, 17).

The authors’ own previous studies have demonstrated that garlic enhances natural killer (NK) cell activity (17) and T-lymphocyte proliferation (18-20). Garlic extract and a garlic protein fraction were shown to augment the oxidative burst in peritoneal macrophages of Balb/c mice (21). Ghazanfari et al. showed that garlic extract induces a shift in cytokine pattern in Balb/c mice with a Leishmania major infection and an upshot in the immune response with regard to Th_1_ (IFN-λ, IL-2) (22, 23). At the same time, a unique garlic preparation called AGE has been reported to have a series of pharmacologic effects including immunomodulation (20). In rodents, AGE and its constituents have been reported to inhibit the development of chemically-induced tumors in the bladder, mammary glands (24, 25), colon, esophagus, lung, skin and stomach (26). Recent studies have focused on the immunological behavior of AGE and AFB_1_ (3). In the present study, the authors investigated the stimulation and suppression of the immune system of Balb/c mice in vitro by AFB_1_ and AEG.

## Materials and Methods

### Materials

#### AFB_1_ preparation

Toxigenic *Aspergillus flavus* (PTCC 5004) was purchased from the Iranian Research Organization for Science and Technology (IROST) and tested for the generation of AFB_1_ by slide chromatography. The *Aspergillus flavus* was then cultured in Aflatoxin production medium to generate mycotoxin. AFB_1_ separated of culture extract by HPLC method (1, 6).

#### Preparation of AGE

Fresh garlic bulbs were obtained from Hamadan, a city in western Iran and famous for its fresh garlic. Dry garlic bulbs were peeled and preserved in the freezer (-20˚C) for six months. Aqueous aged garlic extract was prepared using the Mantis method (20). Garlic bulbs were homogenized with two parts of distilled water in a varying blender. The homogenized blend was filtered under vacuum through Whatman paper (No. 1) and the filtrate was centrifuged at 3400 g for 30 minutes. The clear supernatant was collected. Twenty-seven grams of NH_4_SO4 were added to one liter of the supernatant and centrifuged at 3400 g for 30 minutes. The residue was re-suspended in saline and dialyzed against buffer saline. AGE samples were then run on G 50 gel chromatography to measure protein using the Bradford assay and evaluated by sodium dodecyl sulfate polyacrylamide gel electrophoresis (SDS-PAGE) (24).

#### SDS-PAGE electrophoresis

A 12% (weight/volume) polyacrylamide gel was utilized to judge the purity of molecules and to estimate the molecular mass compacted with proteins. After electrophoresis, the gel was fixated with methanol and acetic acid formaldehyde for 60 minutes and stained with coomassie blue.

#### The sample

The groups of inbred female Balb/c mice age 4-6 weeks were purchased from the Pasteur Institute of Iran. Four groups (five mice in each) were housed in a standard poly-propylene cage. The animals were kept under standard conditions (a cycle of 12/12 hour light/dark and a temperature of 20-22◦C) with free access to water and autoclaved standard mouse chow. Animal care and treatment were conducted in conformity with the guidelines of Animal Care and Research Committee of Tarbiat Modares University and in compliance with the Guide for the Care and Use of Laboratory Animals [DHEW Publication No. (NIH) 85-23, Revised 1985, Office of Science and Health Reports, DRR/NIH, Bethesda, MD 20205].

### Methods

#### Delayed-type hypersensitivity (DTH) test

To evaluate DTH response, 20 female mice were randomly assigned into groups of five. On day 0, 0.1 ml of a solution containing 1×10^8^ sheep red blood cells (sRBCs, Razi Institute, Tehran, Iran) suspended in PBS were subcutaneously injected in the back of all mice. Three groups received three doses (30, 15, 10 µl/kg/ day) of AFB_1_ (0.1 ml) via the intraperitoneal (IP) route over a 5-day period The remaining group (the control group) received diluent [a solution of ethanol /PBS (40:60)] via the same route, dosage, and time interval. On the 5^th^ day, the sensitized animals were subcutaneously challenged with 1×10^8^ sRBCs in the left hind foot pads. The increase in the foot pad thickness was measured with a vernier calliper (Mitutoyo, Japan) one,two and three days after the booster injection of sRBCs. The results were calculated according to the following formula (18, 19):

$$\mathrm{Increased DTH}=\frac{(\mathrm{Left footpad challenged with sRBC}-\mathrm{right footpad})\times 100}{\mathrm{Right foot pad}}$$

#### Preparation of the animal model

The first group in this experimental study was treated with 0.1 ml of AFB_1_ at a daily dose of 10 µl/kg via the IP route. This dose had already been defined by the DTH test (above) as the optimal immunostimulatory dose. The second group (negative control) also received the same volume of diluents PBS via the IP route. The third group was treated with 0.1 ml of AFB_1_ (10 µl/kg/day) and 0.1 ml of AGE (20 mg/kg/day) via the IP route (25) and the fourth group with 0.1 ml of AGE (20 mg/kg/day) via the same route. The treatments were applied once in everyday in 7 day period (26).

#### Splenocyte cytokine production measurement through ELISA method

The isolated spleen mononuclear cells were cultured in 24-well plates (Nunc, Denmark) in a final concentration of 2×10^6^ cells/ml. Three samples were taken from each mouse in the group and each sample was analyzed in triplicate. Twenty microliters of puriﬁed tumor antigen and phythohemagglutinin (PHA) were added to each separately to stimulate the cells.

After three day incubation at 37˚C and 5% CO_2_ , the supernatants were collected and frozen at -70˚C until analyzed by enzyme-linked immunosorbent measurement (ELISA). IFN-λ and IL-4 concentrations were measured using the R&D American DuoSet ELISA Development kit. The treatment mice were unconscious and medullary and seperated spleen.

#### Separation of splenic mononuclear cells (MNC)

The control and treated animals were sacrificed by cervical dislocation on the 13^th^ day; spleens were resected under sterile conditions and were suspended in PBS. The splenic cell suspension was RBC- lysed with 0.75% NH_4_Cl and Tris buffer (0.02%) (pH=7.4). The cells were washed and the single-cell suspension was prepared in RPMI-1640 (Gibco, 51800-035, Stey cell Technology Company) containing stable glutamine (Cytogen, USA) and 10% heat inactivated fetal calf serum (Gibco, England). To define the viability and density of cells in the suspension the Trypan blue dye exclusion method was used. The cells were counted using homocytometer light microscopy. The viability of the splenocytes was generally above 95%. After an additional washing, the suspension was adjusted to 4×10^6^ cells per milliliters in RPMI-1640 supplemented with 10% FCS, 100 µg/mL streptomycin, and 100 IU/mL penicillin (complete RPMI), and kept at 4˚C.

#### Three-color immunostaining and flow cytometry analysis

After treating the mice during the 7-day period as mentioned in 2.2, the MNCs purified from the mice spleens were immunostained with the FITC anti-mouse CD4, PE-Cy5 anti-mouse CD25 (BD, eBioscience, USA), and subsequently with PECy5 anti-mouse FoxP3^+^, according to the eBioscience mouse regulatory Tcell staining kit’s instruction. Three samples were taken from each mouse and each was analyzed in triplicate. The samples were analyzed using a FACSCalibur flow cytometer at Tehran University and the results were analyzed with WinMDI/25 software.

#### Statistical analyses

In this study each experiment was performed in duplicate or triplicate and one-way analysis of variance (ANOVA) or Mann-Whitney non-parametric tests were used to determine the statistical significance (p<0.05) between values in the experimental and control groups. The data were analyzed using SPSS software version 16 and the results are expressed as measures of central tendency and dispersion (mean, SE, etc.).

## Results

### Bradford assay and SDS-PAGE electrophoresis

**Fig 2:**
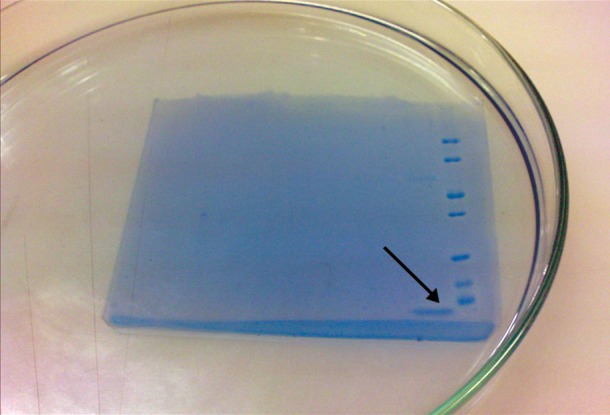
Gel electrophoresis: protein bound in AGE is shown by arrow-head.

The results of the Bradford evaluation showed that the quantity of the effective protein in AGE is 0.27 µg/ml. Gel electrophoresis was performed. The results are shown in figure 2.

### Effect of AFB_1_ on DTH test

**Fig 3:**
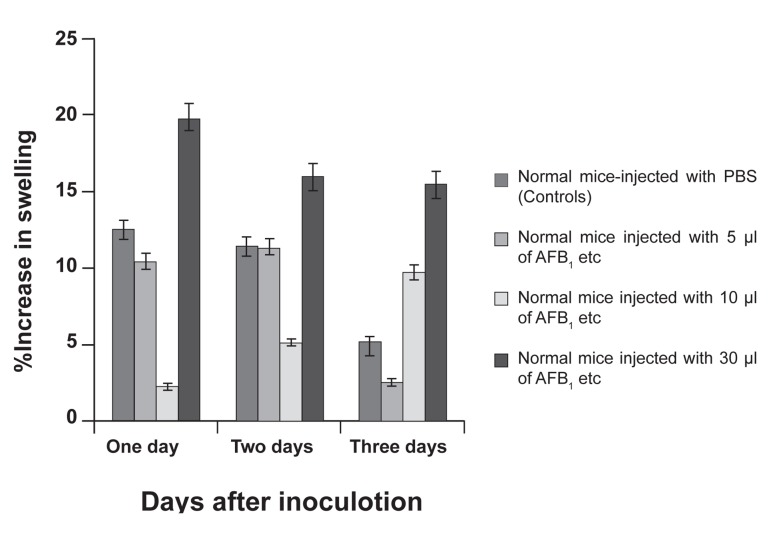
The results of delayed type hypersensitivity (DTH) assay. As shown in the graph a significant difference in the degree of swelling was detected in the group treated with 10 µl of AFB_1_ (p=0.015 and 0.021 at two and three respectively).

In order to estimate the effect of AFB_1_ on cell mediated immunity (CMI), twenty mice (four groups of five mice) were treated with AFB_1_ and PBS as shown in figure 3. For five consecutive days, the mice were sensitized using sRBCs, treated with three doses of AFB_1_ (30, 10, 5 µl/Kg/Day in three groups, and PBS: ethanol 60:40 in the control group). A challenge using sRBCs was then performed in the left foot pad.

The percentage of foot pad swelling was measured using a digital vernier capillier at intervals of one, two and three days. There was a significant difference in mice treated with a dose of 10 µg/kg/day compared to control mice two and three days after the foot pad challenge. The steady increase in the pad swelling two and three days after injection (p value=0.01) showed that a dose of 10 µl/kg/day of AFB_1_ significantly contributed to a greater DTH response every one day after the foot pad challenge compared to controls (p=0.01, 0.046 and 0.021 respectively). This increase was not seen in other groups. As a consequence, the optimum dose of 10 µl/kg/day of AFB_1_ was used for the rest of the investigations.

### Effect of AFB_1_ and AGE on splenic CD4 ^+^ CD25 ^+^ FoxP3 ^+^ T cells

**Fig 4:**
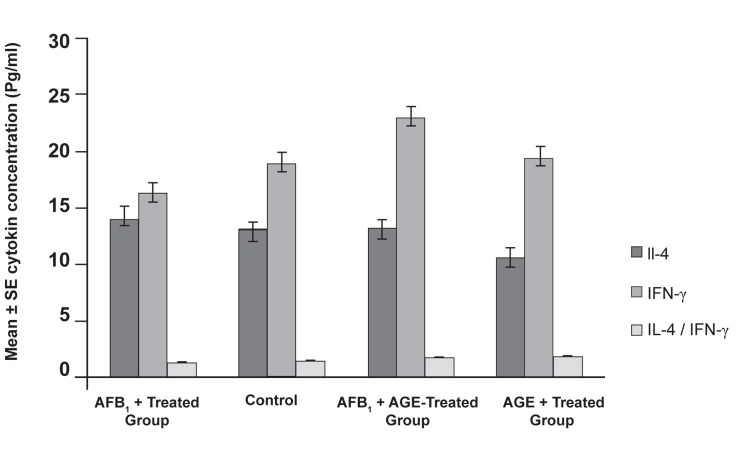
Results of ELISA assessment showing the level of IFN-λ and IL-4 cytokines produced from splenocytes stimulated by specific tumor antigen. Results show a statistically significant difference between the AGE-treated group and AFB_1_-treated group (p<0.05).

**Fig 5:**
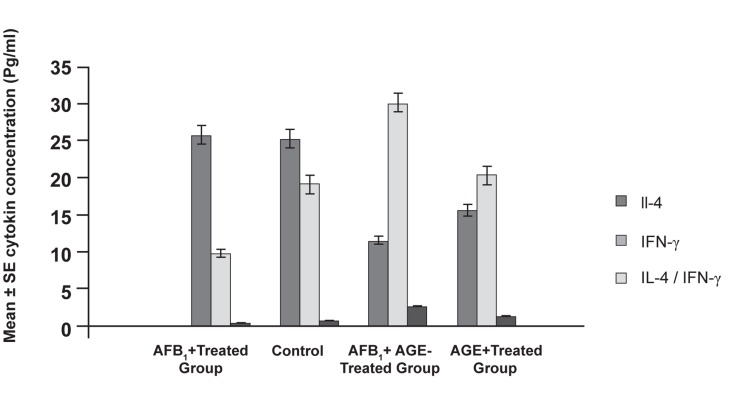
Results of ELISA assessment showing the level of IFN-λ and IL-4 cytokines produced from splenocytes stimulated by specific PHA. Results show a statistically significant difference between the AGE-treated group and AFB_1_-treated group (p<0.05).

Concentration of IFN-λ and IL-4, typical cytokines for Th_1_ and Th_2_ (Previously you described Th_1_ as follows: Th_1_ ((IFN-λ, IL-2)) and Th_2_ pattern has not been explained at all.) pattern, in treated and untreated mice was evaluated by ELISA technique. The results demonstrated that mice treated with AFB_1_ showed a decreased level of IFN-λ and an increased level of IL-4, but in the case of mice treated with AGE, the results showed an increased level of IFN-λ and a decreased level of IL-4. These differences were statistically significant (p<0.05). The results for the IFN-λ and IL-4 concentrations are shown in figure 4 and figure 5.

### Effect of AFB_1_ and AGE on splenic CD4 ^+^ CD25 ^+^ FoxP3 ^+^ T cells

**Fig 6:**
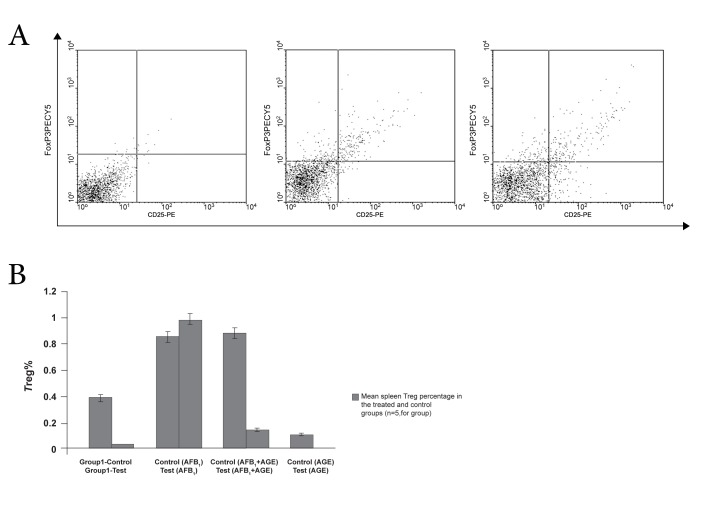
Results of flow cytometry measurement: A. Dot plots representing mean percentage of CD25^+^ and FoxP3^+^ expressing cells on the CD4 + lymphocyte gate of splenocytes. B. Mean ± SE spleen %*T*reg cells in treated and control groups. (p value=0.049).

The flow cytometry technique was used to define the percentage of splenic *T*reg in Balb/c mice. As shown in figure 6 (A and B) the results indicate a statistically significant difference between the percentage of splenic *T*reg cells in the AGE-treated group and the AFB_1_-treated group and the control group and the AFB_1_+ AGE-treated group. The percentage of splenic *T*reg cells in the AGE-treated group was lower than the AFB_1_-treated group (p=0.049).

## Discussion

AFB_1_, a secondary metabolite of *Aspergillus flavus*, has unique chemical structures. These structures react and interact with the chemicals in living organisms causing harm. *Aspergillus flavus* is a hepatocarcinogen in humans and can invade tumor cells. If a human receives a small dose of AFB_1_ on daily basis over a long period of time, it will result in carcinoma (1, 5-7). Unfortunately, it is possible for humans to receive small doses of AFB_1_ while consuming contaminated food, specifically milk. AFB_1_ effects on the immune system can be either stimulatory or suppressive depending on the critical exposure windows of dose and time (9, 10). The immune cells in the spleen, such as T-lymphocytes and macrophages, both significant mediators of inflammatory responses to tissue damage, have been shown to be differently affected by continuous and intermittent exposures to AFB_1_ (11, 12) .

During the past decade, medical researchers have increasingly focused on herbal medicine, especially Garlic. Garlic has been consumed for food and medicinal purposes worldwide for thousands of years. Garlic’s beneficial effects on human health are known to everyone (22, 24). Currently, the garlic plant itself, as well as its numerous extracts, are commercially available as dietary supplements (20, 22, 24). Epidemiological studies suggest garlic consumption has preventive effects in some types of cancer (18). Various researchers have indicated that garlic modulates immune responses (17, 18). Previous studies showed that garlic enhances natural killer cell (NK) cell activity and T-lymphocyte proliferation (19). Also garlic extract and a garlic protein fraction have been shown to augment the oxidative burst in peritoneal macrophages of Balb/c mice (26). Lau et al. showed that AGE is an efficient candidate as an immune modifier compared to fresh garlic extract, which maintains the homeostasis of immune functions (22). In the present investigation, the authors explored the cytotoxity and immunomodulatory activities of AFB_1_ and AGE *in vivo* in a mouse model. First, in order to select the optimal immunostimulatory dose of AFB_1_, the researchers performed DTH measurements. The results demonstrated that a dose of 10 µl/Kg/Day had a significant effect on the DTH test two and three after a foot pad challenge. Based on previous research a dose of 20 mg/kg/Day of AGE were used to perform these tests (17, 18, 20). Our own findings and those of other studies (11, 24-25) found a dose of 10 µl/Kg/Day to be the optimal immunostimulatory dose and this dose was used in all further experimentation. Nevertheless,our results showed that AGE have effects of supporting of immune system. Figure 5 and figure 6 indicate that in AFB_1_-treated groups the level of IFN-λ cytokines decreased in the control group whereas in the AGE-treated groups the level of IFN-λ cytokines increased. Increased production of IFN-λ and decreased IL-4 in turn have other anti-cancer effects including anti-angiogenesis, increased NK activity and increased immune activity. Therefore it seems that a continuous cascade of cytokine production and cellular activation, by definition, explain the AGE anti-cancer mechanisms and increased activation of the immune system. This finding was confirmed in a previous study by Hassan et al. (22) and Noori et al. (23) which indicated that AGE is able to enhance the capacity of splenic leukocytes to produce IFN-λ and IL-4 following mitogenic stimulation. The authors also showed that AGE could decrease and that AFB_1_ could increase the total number of splenic *T*reg cells in the experimental group. Although the mechanism for *T*reg reduction by AGE is not yet fully understood, given that AGE has been shown to decrease tumor growth (24), decrease in the number of *T*reg cells may result in tumor size reduction. Moreover, some studies have reported that AGE can reduce the number of *T*reg cells via the inhibition of NO production "an inducer metabolite for *T*reg expansion" from macrophage cells (21). This finding is confirmed in a previous study by Noori et al. (23) which showed that AGE is able to reduce *T*reg and inhibit tumor growth *in vivo* but AFB_1_ is able to increase *T*reg and stimulation tumor growth *in vivo*.

## Conclusion

Overall, based on the findings of this research and other studies, the authors measured and showed the specific immunomodulatory properties of AFB_1_ that are needed to suppress the immune system and the specific immunomodulatory properties of AGE that are needed to support the immune system. Immune cells in spleen such as T-lymphocytes and macrophages, important mediators of inflammatory responses to tissue damage and cancer, were affected differently by continuous and intermittent exposure to AFB_1_. This study showed that AGE decreases the production of *T*reg cells from splenocytes. Taken together, the findings suggest that AGE may play a role in attenuating tumor growth by increasing cytokine production and decreasing *T*reg cells and anti-tumoral cell activation during cell carcinogenesis. It is possible to speculate that AGE could be used as a plant drug with few side effects during anticancer chemotherapy.
